# Memory processing in neurodevelopmental disorders: linking behavioral, circuits, and molecular scales

**DOI:** 10.3389/fncel.2026.1883287

**Published:** 2026-09-01

**Authors:** Sania Sultana, Sougata Ray, Sarbani Samaddar, Sourav Banerjee

**Affiliations:** National Brain Research Centre, Manesar, India

**Keywords:** ADHD, ASD, intellectual disabilities, memory, neurodevelopmental disorders

## Abstract

Despite long-standing investigations into the neural and molecular correlates of memory, there persists a lack of comprehensive understanding of the cellular and subcellular processes that go awry in cases of neurodivergent and neuropathological phenotypes. While the mechanisms of memory encoding, consolidation, and retrieval are well-characterized in neurotypical models, disruptions in these processes often manifest as cognitive dysfunctions in the contexts of neurodevelopment and neurodegeneration. In this review, we specifically examine the molecular frameworks of memory processing arising from neurodevelopmental disorders. Given that developmental disorders affecting early stages of neural circuitry formation are manifested much later, in the adolescent or adult stages of life, it is important to delineate exactly how the molecular underpinnings of memory formation lead to atypical behavioral phenotypes manifested in different neurodevelopmental conditions. By integrating behavioral phenotypes with underlying circuit and molecular pathologies, this paper argues for a multiscale perspective to understand and address the divergence between neurotypical and neurodivergent memory function.

## 1 Introduction

The ability to remember is one of the most evolutionarily conserved cognitive processes to exist in organismal biology. Memory processing encompasses the involvement of multifactorial mechanisms that emerge from coordinated interactions across behavioral, circuit, and molecular scales. Together, these enable the encoding, consolidation, and retrieval of experience-dependent information. At the molecular level, activity-dependent signaling pathways regulate synaptic plasticity through mechanisms such as long-term potentiation, transcriptional activation, and structural remodeling of synapses, stabilizing memory traces within neuronal ensembles. Distributed networks spanning different areas of the brain, such as the hippocampus, cortex, and subcortical structures, dynamically coordinate to support different memory domains, including working, episodic, and social memory within mammals. These processes are tightly regulated by neuromodulatory systems, which maintain an excitatory–inhibitory balance and ensure network flexibility while maintaining stable information storage.

Disruptions across these interconnected scales can lead to persistent cognitive dysfunction. Increasing evidence suggests that memory impairments across neurodevelopmental disorders arise not from isolated deficits, but from multilevel alterations that span molecular signaling, synaptic plasticity, and large-scale circuit coordination. Conditions such as attention-deficit/hyperactivity disorder (ADHD), autism spectrum disorder (ASD), and intellectual disability (ID) demonstrate distinct yet overlapping memory phenotypes, reflecting diverse underlying mechanisms ranging from neuronal signaling dysregulation to genetic disruptions in synaptic and transcriptional pathways. Examining memory across these disorders provides a transdiagnostic framework for linking behavioral phenotypes with underlying neural mechanisms. Although the neural correlates of memory are affected in neurodegenerative disorders, considerable literature examining the causality and effects of these conditions on memory remains available. In this review, we focus on how memory is affected in disorders stemming from developmental aberrations. A multiscale perspective integrating behavioral outcomes with circuit and molecular processes in the context of neural development is essential for understanding how memory functions in neurotypical and atypical conditions.

## 2 Molecular frameworks of memory formation

**Figure 1:**
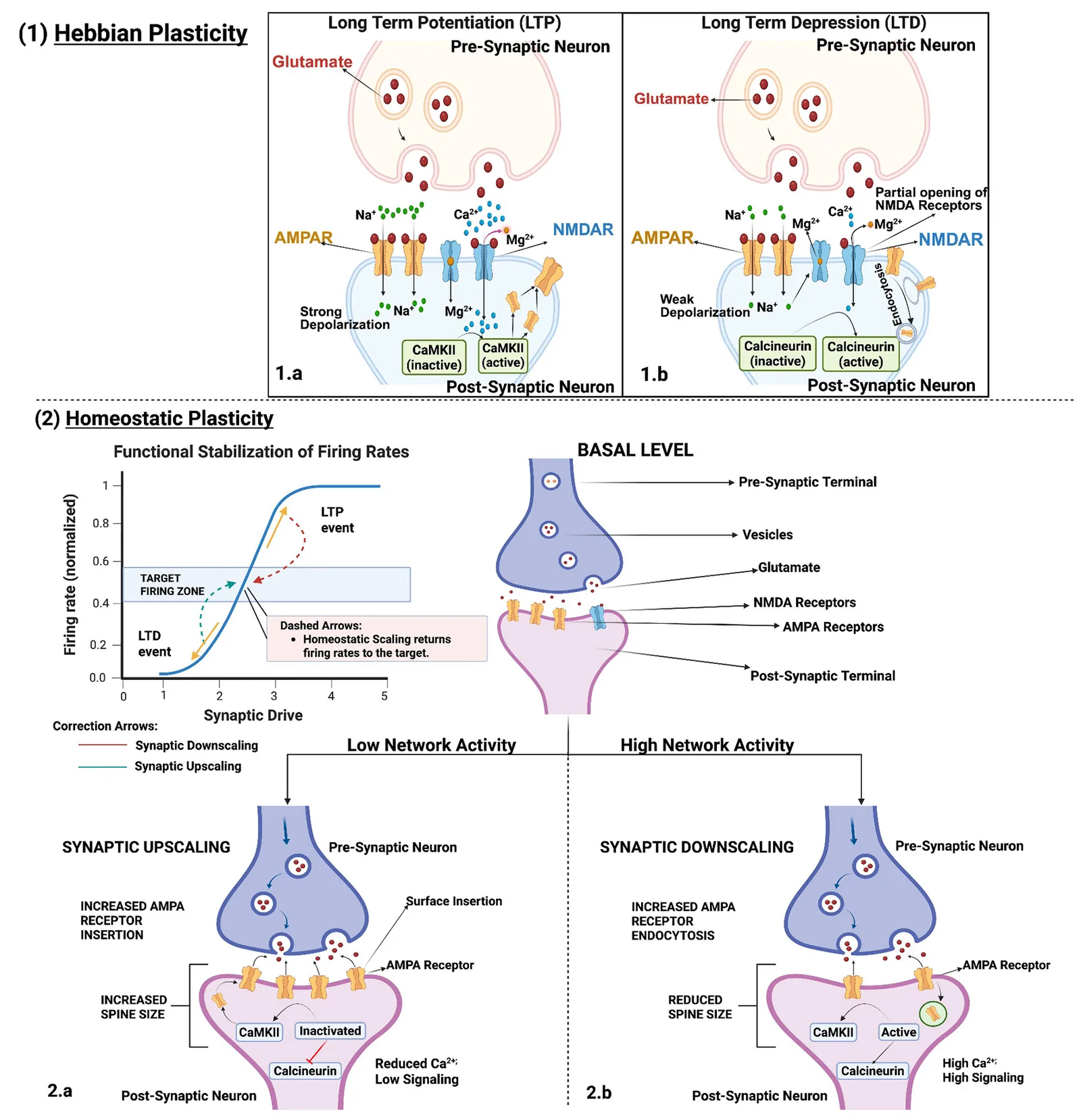
Molecular and circuit level mechanisms of hebbian and homeostatic synaptic plasticity. Hebbian plasticity: incoming synaptic modifications driven by local activity levels. **(1.a)** Long-term potentiation (LTP): postsynaptic depolarization leads to the removal of the Mg^2+^ ion blocking the NMDA receptors. This results in the massive influx of Ca^2+^ ions. This activates the CaMKII leading to the exocytosis and insertion of more AMPA (α-amino-3-hydroxy-5-methyl-4-isoxazolepropionic acid) receptors into the postsynaptic membrane leading to the strengthening of the synapse. **(1.b)** Long-term depression (LTP): weak depolarization results in low influx of Ca^2+^ ions which activates the phosphatases (such as calcineurin). This triggers the endocytosis of the surface AMPA receptors, thereby weakening the synaptic connection. Homeostatic plasticity: overall bidirectional scaling mechanisms that stabilize neuronal firing rates within a target intrinsic zone. The graph represents the functional stabilization of firing rates. An LTP event leads to the firing of neurons above the target zone; this initiates a global synaptic downscaling (shown in red dashed arrow) to restore the baseline. Conversely, a prolonged LTD event drops the firing rate below the target zone leading to the synaptic upscaling (shown in cyan dashed arrow) to recover the normal network activity. **(2.a)** Synaptic upscaling: consistent low network activity promotes synaptic upscaling in individual neurons, leading to the increase in the surface insertion of AMPA receptors from the intracellular pools to scale up the overall synaptic drive. **(2.b)** Synaptic downscaling: following periods of prolonged network hyperactivity, neurons prevent runaway excitation by removing surface AMPA receptors via endocytosis and reduces the synaptic drive, protecting the network and promoting input-specificity (Hebbian LTP/LTD and AMPA receptor trafficking mechanisms adapted from Huganir and Nicoll, 2013 and Lüscher and Malenka, 2012; Homeostatic firing rate stabilization graph adapted from (Turrigiano and Nelson, 2004); molecular scaling mechanisms adapted from Pozo and Goda, 2010).

Memory formation is a dynamic process that emerges from coordinated molecular, synaptic, and circuit-level plasticity. Classical frameworks define memory as the outcome of activity-dependent modifications in neural circuits that encode, consolidate, and retrieve information through long-lasting changes in synaptic strength (Kandel, 2001). Two forms of plasticity—Hebbian plasticity and homeostatic plasticity (Figure 1) play central roles in stabilizing memory representations while preserving network stability.

Hebbian plasticity refers to experience-dependent modifications within specific neuronal ensembles that encode a memory trace. During learning, a subset of neurons is selectively activated and undergoes persistent synaptic strengthening and structural remodeling, forming a distributed engram that helps in later recall (Josselyn and Tonegawa, 2020). These changes are mediated by long-term potentiation (LTP), synaptic tagging, and activity-dependent gene expression involving transcription factors such as CREB, which stabilize synaptic modifications and promote structural plasticity (Samaddar et al., 2023). Engram cells within hippocampal and cortical networks maintain altered synaptic weights and coordinated firing patterns, allowing memory retrieval when reactivated (Tonegawa et al., 2015).

In parallel, homeostatic plasticity ensures stability of neural networks during ongoing learning and synaptic modification. While Hebbian plasticity strengthens or weakens specific synapses, homeostatic plasticity globally regulates neuronal excitability and synaptic strength to prevent runaway excitation or inhibition. This form of plasticity operates over longer timescales than Hebbian plasticity and maintains overall network balance by synaptic scaling, intrinsic excitability adjustments, and regulation of receptor expression while preserving relative synaptic weights that encode memory (Turrigiano, 2012; Srinivasan et al., 2021; Wu et al., 2021). Without such compensatory mechanisms, excessive potentiation or depression would destabilize circuit function and impair information storage.

**Figure 2:**
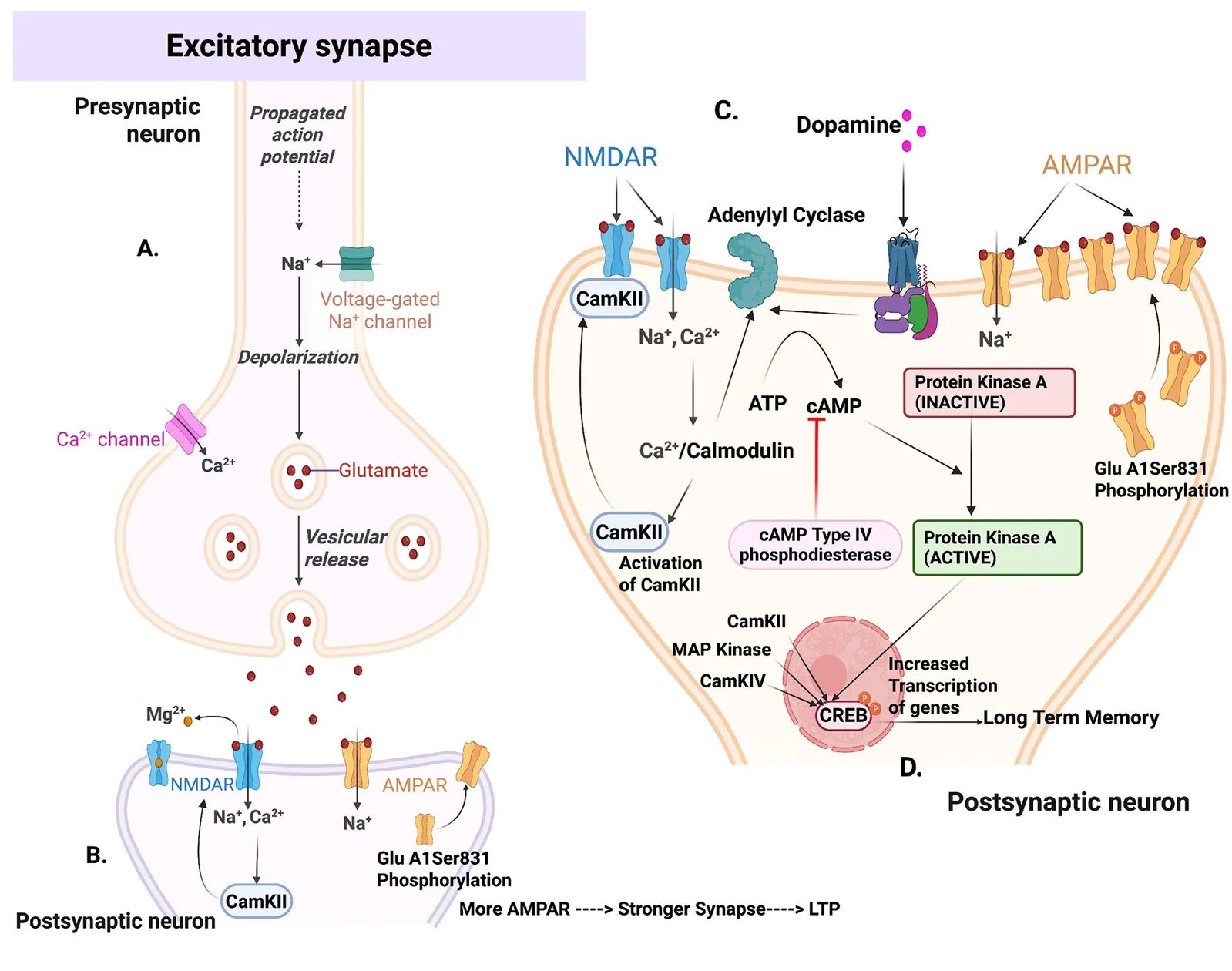
Molecular signaling pathway for long-term potentiation (LTP). **(A)** Induction: propagation of the action potential (facilitated by voltage-gated Na+ channels) depolarizes the membrane, leading to the opening of the voltage gated Ca^2+^ channels. The influx of Ca^2+^ triggers the release of glutamate from the pre-synaptic neuron. The glutamate binds to the AMPA receptor, thereby depolarizing the postsynaptic membrane. The Mg^2+^ ions get removed, thereby causing influx of Ca^2+^ through the NMDARs (Franchini et al., 2020). **(B)** Early-Phase Signaling: Increased levels of Ca^2+^ activate CaMKII, which then binds to the GluN2B subunit of NMDAR (He et al., 2022). CaMKII also phosphorylates the AMPARs (e.g., GluA1 Ser831), leading to the increased recruitment of the AMPARs resulting in stronger synapses and LTP. **(C)** Synergistic activation of adenylyl cyclase: dopamine binds to the D1/D5 receptor. The Adenylyl Cyclase is then activated by the Gs-protein signaling from the D1/D5 receptor, and the Ca^2+^/Calmodulin complex. This leads to the formation of cAMP which in-turn activates the PKA pathway. Excess cAMP is inhibited by Phosphodiesterase (PDE) (Otmakhova and Lisman, 1998; Abel and Lattal, 2001). **(D)** Nuclear transcription: protein Kinase A (PKA) along with CaMKII, CaMKIV, and MAP kinase signaling pathways inside the nucleus, triggers the phosphorylation of CREB. This triggers the transcription of genes which are essential for synaptic plasticity and formation of long-term memory (Abel and Lattal, 2001).

At the cellular level, these processes have traditionally been examined through the lens of synaptic plasticity within excitatory neuronal networks (Figure 2). Most work has focused on activity-dependent plasticity of excitatory pyramidal neurons within hippocampal and cortical circuits, as these neurons form the principal substrates for engram formation and long-term memory storage. However, emerging evidence highlights the essential contribution of inhibitory neurons in shaping memory processing. GAB Aergic interneurons regulate the timing and synchrony of excitatory activity, thereby influencing neuronal ensemble recruitment during encoding and retrieval (Buzśaki and Wang, 2012; Lovett-Barron et al., 2014). Together, inhibitory networks maintain excitatory–inhibitory balance and stabilize memory engrams across distributed neural systems.

Disruption of this excitatory–inhibitory balance is increasingly recognized as a central mechanism underlying memory dysfunction across neurodevelopmental disorders (NDD), providing a framework for linking molecular and circuit-level alterations to behavioral phenotypes. Alterations at the molecular, synaptic, or circuit-level can result in persistent cognitive and behavioral impairments. Examining memory dysfunction across NDDs, therefore, provides a critical framework for linking behavioral phenotypes with underlying circuit and molecular mechanisms.

## 3 Neurodevelopment disorders and memory

NDDs are characterized by early-onset alterations in brain development that affect cognition, behavior, and adaptive functioning. Across these conditions, memory dysfunction emerges as a common but heterogeneous phenotype, reflecting disruptions in synaptic plasticity, circuit maturation, and cellular signaling (Parenti et al., 2020). Examining memory across NDDs provides a transdiagnostic framework for linking behavioral impairments with underlying molecular and circuit-level mechanisms. A consolidated overview of the behavioral, circuit, and molecular mechanisms underlying memory dysfunction discussed in this review is presented in Table 1.

**Table 1:** Summary of behavioral, circuit-level, and molecular mechanisms underlying memory dysfunction across neurodevelopmental disorders.

Sl. no.	Reference	Model	Organism	Gender	Memory type	Region of deficit	Mechanism	Result/key findings
1	Meng et al., 2023	Auts2 del. (ASD)	Mouse	Male	Social memory	Dentate gyrus (DG)	↑ oxytocin → ↑ newborn neurons in DG.	EE—-> ↑ oxytocin; ↑social novelty & ↑ memory.
2	Choi et al., 2024	16p 11.2 del. (ASD)	Mouse	Male	Object recognition	Locus coerulus system	Spatial object recognition (SOR) was performed; deficit in LC-NE neurons	Rescue: Inhibition⊣ LC-NE neurons reverse memory in 16p11.2 mouse.
3	Çetinkaya et al., 2025	NLGN1/NLGN2 (ASD)	Mouse	-	Spatial Memory	Hippocampus	disrupted cotricostriatal signaling	↓ Spatial memory; impaired hippocampal LTP
4	Guo et al., 2023	Dock4 (ASD)	Mouse	Male	spatial memory	Hippocampal dysfunction	Barnes maze test; ↓mature neurons; ↓oligodendrocytes; ↑astrocytes.	↓ spatial memory.
5	Coutelle et al., 2021	ASD	Human	Male & Female	Episodic memory	Connectivity deficit	spontaneous recall to the cueing phase	↓ retrieval of memory.; Semantic memory intact.
6	Clement et al., 2012	Syngap +/- (ASD)	Mouse	Not Specified	Working/ remote memory	Dentate Gyrus	Syngap1 negatively regulates Ras signaling pathway & AMPA receptor insertion into the post-synaptic membrane	↓ Working Memory; ↓ Remote Memory.
7	Chen et al., 2024	ASD	ASD-Human & Non-ASD- Human.	Male & Female	Episodic/ visual memory.	Hippocampus & Ventral Temporal lobe.	Circuit level alterations; Molecular mechanisms not specified	↓ connections in ASD humans, But with ↑ Age → ↓ connection in ASD & Non-ASD humans.
8	Heitz et al., 1992	FMRI (FXS)	Mouse	Not specified	Long-term memory; short-term memory; emotional memory	Hippocampus	Long-Term Hippocampus dependent Memory in Morris Water Maze Test; Novel Object recognition Task; ↑ inhibitory avoidance extinction	↓ Acquisition and Reversal Memory.; ↓ Short-Term Memory.; Impaired Emotional Memory.
9	Li et al., 2020	Fmr1 KO (FXS)	Mouse	Male	Fear memory recall	Hippocampus	Defective memory engram reactivation	Impaired fear memory recall due to failure of memory engram reactivation
10	Neul et al., 2010	MECP2 (Rett)	Humans, predominantly female	Female (predominant); Male	Learning and memory impairments; cognitive deficits	Hippocampus	MECP2 mutations causing neurodevelopmental dysfunction	Impaired synaptic plasticity.
11	Wang et al., 2023	MECP2 (Rett)	Mouse	Female			circuit level intervention	partial rescue of memory stability.
12	Asaka et al., 2006	Mecp2-null	Mouse	Male	Spatial Memory; Contextual Memory	Hippocampus	Impaired hippocampal LTP	Deficits in synaptic plasticity associated with memory impairment
13	Chao et al., 2010	Mecp2 mutant (Rett)	Mouse	Male	Learning and Memory	Hippocampal and Cortical Circuits	GABAergic dysfunction; altered excitatory–inhibitory balance	Network dysfunction and impaired information processing contributing to cognitive deficits
14	Williams et al., 2006.	UBE3A	Human Both sexes	Male & Female	Working memory; learning; long term memory.	Hippocampal circuit.	Deficit in associative learning;	↓ Working memory; ↓ Long-term memory.
15	Sato and Stryker, 2010	UBE3A	Ube3a-deficient mouse	not specified	Experience-dependent plasticity.	Visual cortex	Impaired synaptic plasticity and experience-dependent cortical remodeling	Loss of UBE3A disrupts normal cortical plasticity mechanisms
	Su et al., 2023	UBE3A	Ube3a-deficient (Angelman syndrome) mouse	Not specified	Hippocampus-dependent learning and memory	Hippocampus	Defective synaptic plasticity associated with UBE3A deficiency	UBE3A loss leads to impaired learning and memory and altered hippocampal plasticity.
	Sun et al., 2015	UBE3A	Angelman syndrome mouse	Both sexes	Contextual fear memory	Hippocampus	Altered activity-dependent transcriptional responses during memory recall	UBE3A deficiency disrupts hippocampal gene expression programs engaged during fear-memory retrieval
16	Baddeley and Jarrold, 2007; Carney et al., 2013; Godfrey and Lee, 2018; Lanfranchi et al., 2012; Smigielska-Kuzia et al., 2011; Koenig et al., 2021	Downs Syndrome	Human	Both sexes	Working Memory; Episodic Memory	Hippocampus; Pre-Frontal Cortex.	Disruption in Hippocampal-pre-frontal circuitry; impairment of encoding;	↓ Memory Retrieval.
17	Wang et al., 2012	DSCR1 overexpression (Down Syndrome)	Mouse	Not specified	Learning and Memory	Hippocampus	DSCR1–FMRP interaction; dysregulated local protein synthesis; altered spine morphogenesis	Impaired dendritic spine development and activity-dependent local protein synthesis
18	Al-Saad et al., 2021; Phillips et al., 2025	ADHD (clinical subtypes)	Human	Both sexes	Working memory (WM); learning; delayed recall	Prefrontal cortex	Executive dysfunction	↓ WM; ↓ verbal & visual delayed recall; impacts learning & goal-directed behaviour
19	Cubillo et al., 2011; Wolf et al., 2009	ADHD (fMRI)	Human	Both sexes	Working memory; interference control	Prefrontal–striatal–cerebellar circuits	Circuit hypoactivation	↓ DLPFC/VLPFC activation; ↓ fronto-striatal activity; altered cerebello- prefrontal coordination
20	Mereu et al., 2017	DAT hypofunction	Mouse	Not specified	Working memory; learning	Prefrontal–striatal circuits	Dopaminergic dysregulation	↓ dopamine signaling → hyperactivity & ↓ learning
21	Miller et al., 2014	Glutamatergic dysfunction	Rodent	Not specified	Working memory; goal maintenance	Prefrontal cortex	↓AMPAR transmission	↓ prefrontal excitatory transmission → ↓ WM & executive control; rescue restores cognition
22	Fallgatter et al., 2013; Regan et al., 2019; Bacanli et al., 2021	LPHN3, DAT1, DRD4 variants	Human + rodent	Both sexes / mixed	Working memory; cognitive control	Prefrontal–striatal circuits; hippocampus	Genetic modulation of neuromodulation	↓ prefrontal engagement; ↓ cognitive control; ↓ hippocampal plasticity;
23	Tibu et al., 2016b; Sterley et al., 2016	Early adversity / maternal deprivation	Human + rodent	Both sexes / mixed	Working memory; spatial memory	Prefrontal–limbic circuits	Developmental catecholamine disruption	Early stress → ↓ WM & response inhibition; ↓ spatial memory;
24	Ehninger et al., 2008	Tsc2+/–	Mouse	Male	Learning and memory	Hippocampus	Hyperactive mTOR signaling; impaired LTP	Learning deficits rescued by mTOR inhibition
25	López-Aranda et al., 2024	Tsc2+/– (TSC)	Mouse	Male and female	Object memory; social memory	Hippocampal circuits	Sex-dependent effects of Tsc2 haploinsufficiency	Female mice exhibit object memory deficits; males exhibit social memory deficits

Symbols: ↑ (Increase / Upregulation); ↓ (Decrease / Downregulation); → / → (Leads to / Results in); ⊣ (Inhibits / Blocks). Abbreviations: ADHD, attention-deficit/hyperactivity disorder; AMPAR, AMPA receptor; ASD, autism spectrum disorder; DAT, dopamine transporter; DG, dentate gyrus; DLPFC, dorsolateral prefrontal cortex; DSCR1, down syndrome critical region 1; EE, enriched environment; fMRI, functional magnetic resonance imaging; FMRP, fragile x messenger ribonucleoprotein; FXS, fragile x syndrome; KO, knockout; LC-NE, Locus coeruleus-norepinephrine; LTP, Long-term potentiation; mTOR, mammalian target of rapamycin; SOR, spatial object recognition; TSC, tuberous sclerosis complex; VLPFC, ventrolateral prefrontal cortex; WM, working memory.

### 3.1 Attention-deficit hyperactivity disorder

**Figure 3:**
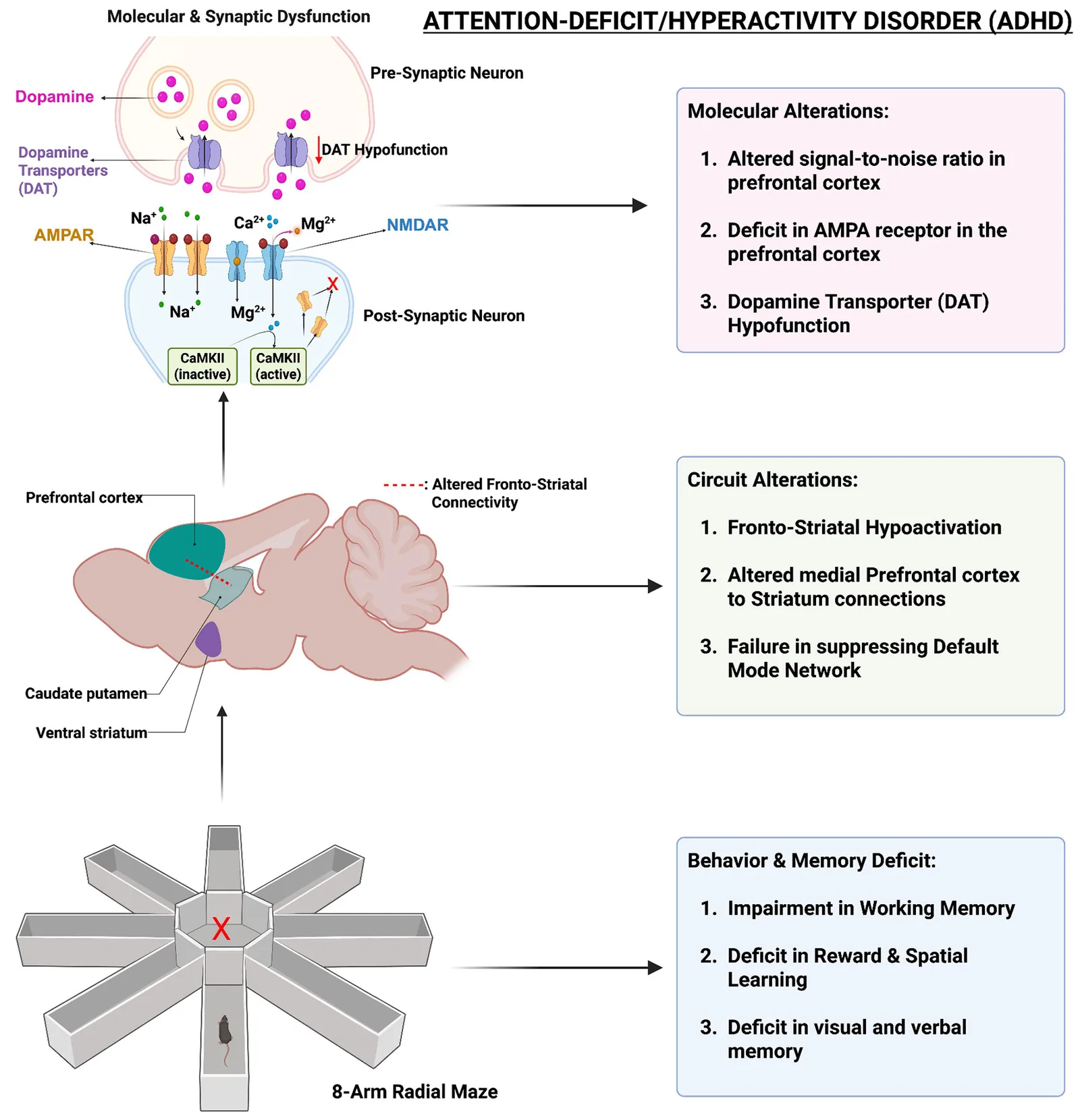
Multiscale pathophysiology of attention-deficit/hyperactivity disorder (ADHD): linking molecular and circuit dysfunction to behavioral deficits. **(Top)** Molecular & Synaptic Dysfunction: ADHD memory dysfunction is primarily regulated by disruption in the neurochemical transmission. Dopamine Transporter (DAT) hypofunction and impaired AMPA receptor signaling in the prefrontal cortex, disrupt the signal-to-noise ratio which is required for context-dependent working memory & executive control. **(Middle)** Circuit alterations: These cognitive impairments are caused primarily due to the dysfunction within the prefrontal-striatal circuitry. This includes hypoactivation of the fronto-striatal network during working memory tasks, altered medial prefrontal cortex-striatum connectivity (including the ventral striatum, indicated by the red dotted line), and a failure to suppress the Default Mode Network (DMN) during goal directed behaviors. **(Bottom)** Behavior & Memory Deficits: ADHD is well characterized by severe impairments in working memory, reward/spatial learning, and delayed visual and verbal recall. Deficits in retention, spatial navigation, & executive control in different preclinical models of ADHD are assessed using behavioral paradigms such as 8-arm radial maze. (Adapted from Zhu et al., 2010; Al-Saad et al., 2021; Phillips et al., 2025; Wolf et al., 2009; Cubillo et al., 2011; Brown et al., 2011; Oades et al., 2005; Miller et al., 2014; Mereu et al., 2017).

As memory dysfunction emerges as a key cognitive impairment, ADHD provides a well-studied model for understanding how disruptions in circuit coordination and neurotransmitter balance affect memory processing across development. This section reviews evidence showing that memory dysfunction in ADHD arises from disrupted neurotransmitter signaling, not gross structural pathology. Attention-deficit hyperactivity disorder (ADHD) is one of the most prevalent neurodevelopmental disorders characterized by disruptions in attention, executive control and regulation. Memory dysfunction in ADHD occurs due to the disruption of working memory and executive control. Across development, these deficits in retention and information modulation directly impair learning and goal-directed behavior (Al-Saad et al., 2021). The multiscale mechanisms underlying memory dysfunction in ADHD are summarized in Figure 3.

Recent human studies show that ADHD individuals exhibit pronounced disruptions in learning and delayed recall of verbal and visual information (Phillips et al., 2025). Furthermore, developmental adversity also plays an important role in memory in learning, as shown by research on early institutionalized children. They exhibit poor working memory and response inhibition that partially mediate later symptom severity (Tibu et al., 2016b,a). Rodent models of maternal deprivation exhibit long-lasting impairments in spatial learning and memory, as well as altered limbic circuitry, suggesting that early catecholaminergic disruption contributes to later cognitive dysfunction (Zhu et al., 2010; Sterley et al., 2016). ADHD is also associated with deficits in delay-dependent learning and odor memory, suggesting disrupted attention-dependent encoding rather than primary storage failure (de Meyer et al., 2019; Miller and Castel, 2025; Peng et al., 2025).

Neuroimaging studies link these behavioral deficits to aberrations in the prefrontal–striatal circuits. Functional MRI studies demonstrate dorsolateral and ventrolateral prefrontal cortex hypoactivation during working memory tasks. The circuit-level dysfunction is also highlighted by the reduced activation of fronto-striatal during interference inhibition and attention allocation tasks in adults. Furthermore, these abnormalities extend beyond the fronto-striatal loops. Altered cerebellar involvement and asynchronous cerebello–prefrontal circuits during working memory tasks point to cerebellum as a regulatory hub contributing to memory processing in ADHD (Wolf et al., 2009; Cubillo et al., 2011).

At the molecular level, dysregulated neurochemical transmission is the primary reason for memory dysfunction in ADHD. In the prefrontal circuits, signal-to-noise regulation is disrupted by impaired dopamine and norepinephrine signaling, disrupting context-dependent working memory (Oades et al., 2005). Furthermore, the fronto-striatal circuit development is also affected by dopamine transporter (DAT) hypofunction (Mereu et al., 2017). In the prefrontal cortex, AMPA receptor–mediated glutamatergic dysfunction also contributes to deficits in working memory and goal maintenance (Miller et al., 2014).

Genetic studies demonstrate that alterations in cellular signaling and circuit development play an important role in memory and executive dysfunction in ADHD. For instance, in humans, LPHN3 risk haplotypes have altered cognitive response control and prefrontal engagement, whereas rodent knockouts exhibit increased activity, hippocampal memory deficits, and impaired synaptic plasticity (Fallgatter et al., 2013; Regan et al., 2019). DAT1 variants tip the balance between task-positive executive networks and default mode suppression, influencing working memory (Brown et al., 2011; Bacanli et al., 2021). DRD4 polymorphisms are linked to differences in verbal working memory as a heritable endophenotype (Loo et al., 2008; Yin et al., 2014; Tian et al., 2024). Additional risk loci (CDH13, DCDC2, ATXN1–CIC, TRIM31) converge on disrupted circuit maturation, linking genetic susceptibility to memory and executive control deficits in ADHD (Tian et al., 2024).

Collectively, memory dysfunction in ADHD is influenced by both genetic and environmental factors, influencing neuromodulatory control and large-scale circuit coordination. Areas involving attention and prefrontal executive systems are impacted, rather than gross structural synaptic pathology, with memory emerging as a unifying phenotype linking behavior, circuits, and molecular mechanisms.

### 3.2 Autism spectrum disorder

While ADHD mainly revolves around the volatility of attention and regulation, another aspect of neurodevelopmental disorders (NDDs)- autism spectrum disorder (ASD), is highly heterogeneous and illustrates how alterations in circuit integration and sensory–cognitive processing shape memory formation and retrieval. Due to this high heterogeneity and complex etiology of NDDs, there is an increase in the transdiagnostic approach mainly focused on behavioral aspects or physical symptoms rather than primary diagnosis (Pohl and Hörnberg, 2022). With respect to this, ASD is mainly involved in deficits in memory, cognition, behavior, and other social domains (Cooper and Simons, 2019).

Furthermore, clinical data reveals a pronounced sex bias in ASD, with males being diagnosed approximately four times more frequently than females (Loomes et al., 2017). This variance is largely attributed to a “female protective effect,” a model which suggests that females require a larger burden of genetic mutations or environmental factors to manifest the same cognitive, social, and behavioral phenotypes as their male counterparts (Jacquemont et al., 2014).

Memory is the central part of every individual and plays a major role in development, learning, and social communication (Cooper and Simons, 2019). ASD individuals remembered fewer internal details about an actual event compared to non-autistic individuals (Coutelle et al., 2021). In some ASD individuals, episodic recollection is disproportionately impaired, while other forms, such as semantic, implicit, and familiarity-based recognition memory remain intact. This separation is evident in both temporal processing, where individuals retain general knowledge but have difficulty recalling the order of events, and in self-identity, where semantic knowledge is intact despite deficits in retrieving specific episodes. ASD individuals also show deficits in episodic memory, which acts as a major center for processing sensory, emotional, and perceptual details that fail to bind together (Cooper and Simons, 2019).

The use of transgenic mouse models has been a very effective and accessible mode for studying NDDs. Transgenic mouse models with specific gene knockout have the capability to replicate the specific genetic risk factors associated with the disorder and reveal the very precise mechanisms that drive the circuitry failure, which in turn is responsible for NDDs. For example, Dock4 knockout mice displayed disrupted object location memory and an age-related specific decline in spatial memory. A decrease in mature neurons and oligodendrocytes, but increased astrocytes in the hippocampus of late-middle-aged mice has been observed in cases of Dock4 deficiency (Guo et al., 2023). Similarly, studies on other models, such as 16p11.2 deletion mice, showed impaired memory in Spatial Object Recognition (SOR) tests. Also, inhibiting Locus Coeruleus-Norepinephrine (LC-NE) neurons reversed this memory impairment (Choi et al., 2024). Furthermore, the loss of the gene in the Auts2 deletion model caused physical hypoplasia of the Dentate Gyrus. This defect is directly responsible for the inability to recognize social novelty, proving that the disruption of a single gene can disrupt a structure, thereby leading to the impairment of social novelty recognition (Meng et al., 2023).

**Figure 4:**
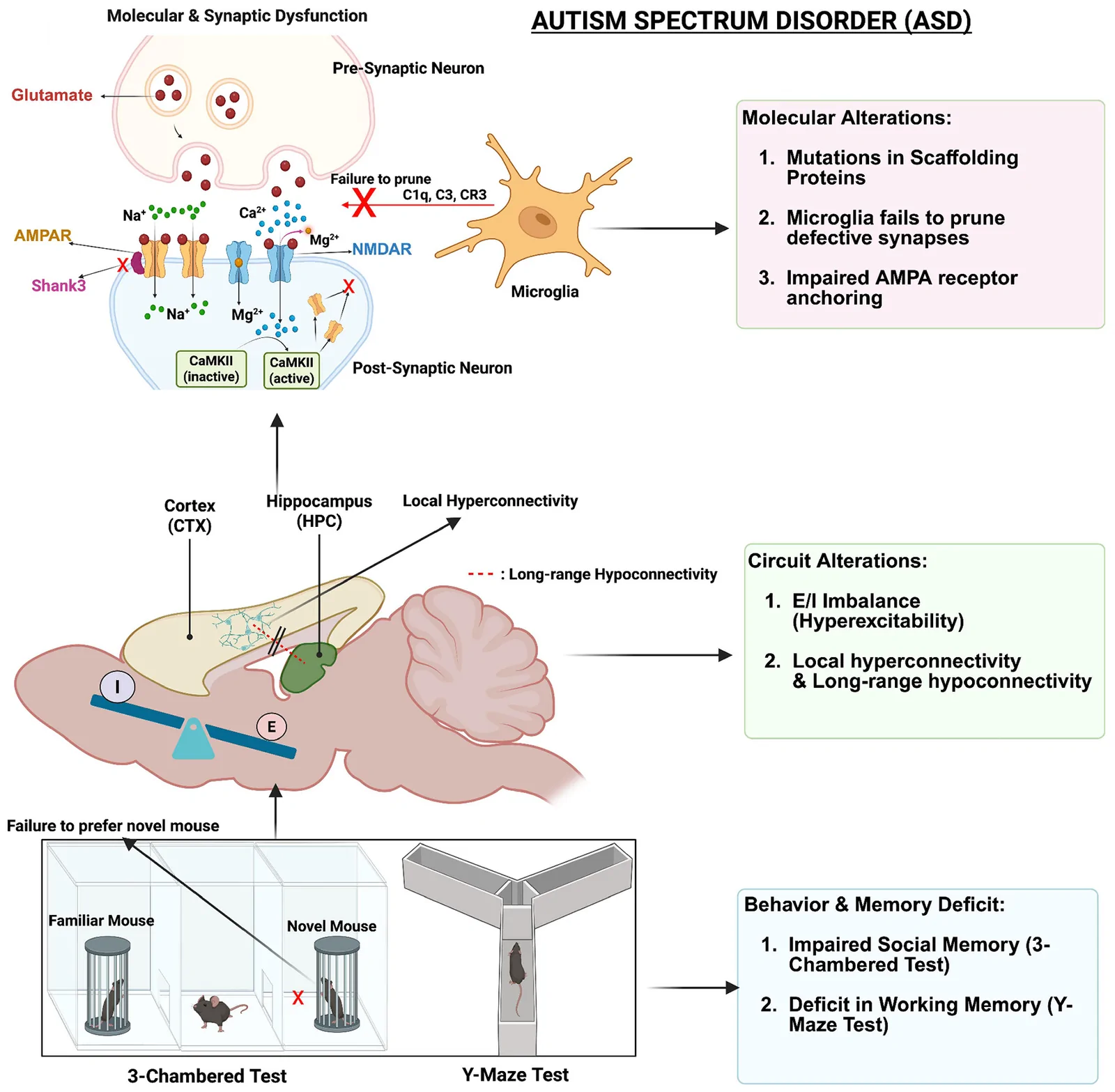
Multiscale integration of autism spectrum disorder (ASD): linking molecular and circuit dysfunction to behavioral deficits. **(Top)** Molecular & Synaptic Dysfunction: The altered circuitry at the synaptic level is caused by different mutations in core scaffolding proteins (such as Shank3), which lead to the disruption in the stabilization of the AMPA and NMDA receptors, which is required for plasticity. Simultaneously, microglia also fail to prune weak and immature synapses during development (indicated by the red X) which leads to structural rigidity and hyperexcitability. **(Middle)** Circuit Alterations: The impairment in cognition and social memory is due to the severe excitatory/inhibitory (E/I) imbalance. Cortical hyperexcitability leads to higher internal neural noise (local hyperconnectivity, depicted by the dense neural network in the CTX), which further disrupts the synchronized communication between the cortex and the hippocampus; this long-range hypoconnectivity is indicated by the red dotted line. **(Bottom)** Behavior & Memory Deficits: Different preclinical mouse models of ASD show prominent deficits in social memory and social novelty recognition. This is assessed via the 3-chambered test, where the ASD model fails to prefer the novel mouse over the familiar mouse (indicated by the red X). This is accompanied by deficits in spatial and working memory (assessed via the Y-maze test). (Adapted from Zhan et al., 2014; Monteiro and Feng, 2017; Belmonte et al., 2004; Meng et al., 2023; Choi et al., 2024).

The altered circuit at the molecular level is primarily driven by the alteration in synaptic scaffolding and developmental refinement, which is further reflected in different behavioral phenotypes. Mutations in core synaptic scaffolding proteins, specifically the SHANK family (SHANK1, SHANK2, SHANK3), account for approximately 1% of ASD. The SHANK family proteins act as organizers within the postsynaptic density (PSD) of excitatory glutamatergic synapses. They act as synaptic scaffolds and connect the membrane-bound neurotransmitter receptors to the actin cytoskeleton via different proteins like PSD-95, Homer etc. Thus, deletion of the SHANK genes leads the PSD to lose essential proteins required to maintain the synaptic scaffold, thereby leading to altered dendritic spine size, altered spine density and failure to anchor postsynaptic AMPA and NMDA receptors, which is essential for Hebbian long-term potentiation (LTP). Different studies showed that restoring SHANK3 gene can reverse some, though not all, ASD-like phenotypes (Monteiro and Feng, 2017). Other molecules such as neurexins (e.g., NRXN1) and neuroligins (e.g., NLGN3, NLGN4X) are presynaptic and postsynaptic cell adhesion molecules that are primarily responsible for the strengthening of the synapse, shaping synaptic efficacy and specifying circuit properties. In ASD, these proteins are heavily compromised by mutations, and that leads to the disruption of the structural integrity of the synapse and disrupts the excitatory/inhibitory (E/I) balance, which further leads to the functional loss of the synapse (Südhof, 2008). Mutations in intracellular signaling proteins like SYNGAP1 cause early maturation of the dendritic spines, triggering severe neural hyperexcitability and an E/I imbalance (Clement et al., 2012). Microglia prune the weak and immature synapses during early brain development for proper brain circuit maturation. Disruption in microglial-mediated synaptic pruning prevents the removal of the weak and unwanted connections during the developmental period (Zhan et al., 2014). This microglial-mediated pruning heavily implies the classical complement cascade, utilizing immune proteins such as C1q and C3 to tag weak synapses, which are thereby recognized and engulfed via microglial receptors like CX3CR1 and CR3 (Paolicelli et al., 2011). Together these molecular alterations lead to a profound E/I imbalance primarily characterized by cortical hyperexcitability, which creates an intense neural noise which leads to the disruption of the long-range connectivity between the prefrontal cortex and the hippocampus, which ultimately culminates in the spatial and working memory deficits observed clinically (Belmonte et al., 2004). The comprehensive multiscale progression of ASD, from core synaptic and microglial dysfunctions to macroscopic circuit alterations and subsequent behavioral memory deficits is visually summarized in Figure 4.

The chronic hyperexcitability observed in ASD individuals points to something going wrong at a much deeper level, a failure of the brain's own internal regulation system. In a typical brain, when activity stays elevated for too long, the network essentially knows to bring itself back down. It does this through a process called homeostatic downscaling, where the Arc/Homer1a pathway gradually pulls AMPA receptors off synapses and brings firing rates back to a manageable baseline (Diering et al., 2017). In an autistic brain, the circuit appears to be seriously broken (Nelson and Valakh, 2015). The network simply can't scale down its own overactivity, so the excess excitation just stays there, uncompensated, and the whole circuit grows increasingly rigid over time. The cost of this is enormous. Without anything to preserve the signal-to-noise ratio, sensory and cognitive inputs stop being processed cleanly. The brain struggles to flexibly update what it's already learned or shift its responses when the situation demands it. Thus, the behavioral pull toward rigid, repetitive thinking finds its neurological roots (Bourgeron, 2015).

Importantly, emerging evidence suggests that these disruptions remain plastic and amenable to intervention. Studies have shown that changing the environment—such as by pairing with a new companion—can rewire the brain's circuitry and restore memory function (Yang et al., 2024). Studies have shown that interventions like repeated oxytocin administration and environmental enrichment helped in the improvement of memory and increased social novelty. Additionally, genetic modification-based neural stem cell expansion improved impaired social novelty recognition (Meng et al., 2023). Together, these findings indicate that ASD memory deficits are not necessarily permanent; with the appropriate environment or therapeutic drugs, the deficits may be modifiable through circuit- and molecular-level interventions.

### 3.3 Intellectual disability (ID)

**Figure 5:**
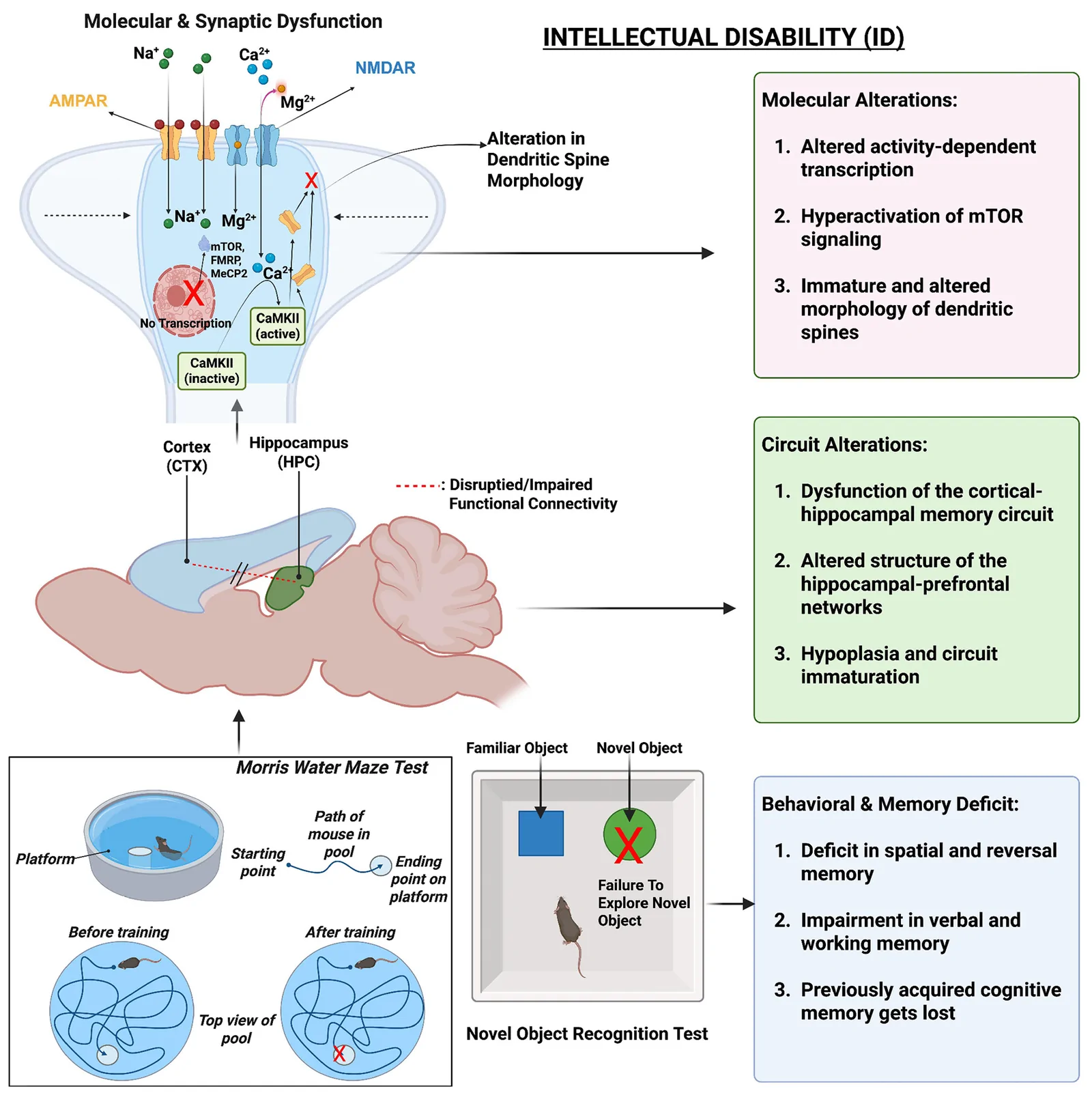
Multiscale pathophysiology of intellectual disability (ID): linking molecular and circuit dysfunction to behavioral deficits. **(Top)** Molecular & Synaptic Dysfunction: The altered circuit networks are primarily driven by disrupted activity-dependent transcription (e.g., FMRP or MeCP2 loss), hyperactivation of mTOR signaling and altered dendritic spine morphology. These defects alter both Hebbian and homeostatic plasticity, thereby preventing the normal synaptic maturation and circuit consolidation. **(Middle)** Circuit Alterations: Behavioral alterations primarily arise from the functional dysfunction within the cortico-hippocampal and hippocampal-prefrontal networks (red dotted line denotes disrupted/impaired functional connectivity). Immature circuits, along with altered excitatory/inhibitory balance (E/I), lead to altered information processing and abrupt network synchronization. **(Bottom)** Behavior & Memory Deficits: Different animal models of ID exhibit severe impairments in spatial and reversal memory (assessed via Morris Water Maze Test) & short-term recognition memory (Novel Object Recognition Test). These reflect the core disruptions in working memory, executive control and loss of previously acquired cognitive abilities. (Adapted from Alloway, 2010; Neul et al., 2010; Verma et al., 2019; Chao et al., 2010; Feliciano, 2020; Koenig et al., 2021; Murphy et al., 2024; Ehninger et al., 2008; Santoro et al., 2012; Tillotson and Bird, 2020).

Beyond ADHD and ASD, memory dysfunction is also a central feature of intellectual disability (ID), where impairments often reflect early developmental disruption of synaptic plasticity and circuit maturation rather than primarily attentional or regulatory deficits. ID is a heterogeneous neurodevelopmental condition characterized by early-onset limitations in intellectual functioning and adaptive behavior. Beyond global learning difficulties, memory impairment is a prominent and clinically meaningful feature of ID, with consistent involvement of working memory (WM) and executive control processes (van der Molen et al., 2007; Alloway, 2010; Schuchardt et al., 2011) that support efficient encoding, maintenance, and goal-directed retrieval of information (Danielsson et al., 2010). Unlike ADHD, where memory deficits are influenced by attentional state and signal transmission, memory dysfunction in ID often reflects developmental disruption of synaptic plasticity and circuit maturation, which persists across development and affects multiple cognitive domains (Alloway, 2010; van der Molen et al., 2009). The multiscale progression, from core molecular and synaptic dysfunctions to disrupted circuit networks and the resulting cognitive and memory deficits is visually summarized in Figure 5.

#### 3.3.1 Fragile X syndrome (FXS)

Fragile X Syndrome (FXS) is a neurodevelopmental disease which is caused due to the overexpansion of CGG repeat (>200 nucleotides) within the 5′-untranslated region (UTR) in the Fragile X gene. This results in the methylation and transcriptional silencing of the FMR1 gene (Kwon et al., 2001; Menon et al., 2004; Vinueza Veloz et al., 2012). It is an X-linked chromosomal disorder which generally presents with more severe cognitive impairments in males than females, reflecting differences in residual FMRP expression resulting from X-chromosome inactivation (Santoro et al., 2012; Li et al., 2020). FXS is the most prevalent monogenic form of inherited intellectual disability (Wang et al., 2018). Previous studies have shown that FMR1 KO mice show impairment during the phases of acquisition, and reversal memory during Morris Water Maze test. FMR1 KO mice have deficits in short-term memory during novel-object recognition tasks. FMR1 KO mice also demonstrated impaired emotional memory processing (Verma et al., 2019).

These behavioral deficits are associated with dysfunction of hippocampal circuits involved in memory encoding, consolidation, and retrieval. Loss of FMRP disrupts activity-dependent translational control of numerous synaptic mRNAs, leading to excessive protein synthesis, altered synaptic maturation, and dysregulated pathways that are critical for learning and memory (Santoro et al., 2012). Consistent with these molecular abnormalities, FXS models exhibit defects in synaptic plasticity, including enhanced mGluR-dependent long-term depression and impaired homeostatic plasticity arising from disrupted FMRP-dependent regulation of activity-dependent protein synthesis, (Santoro et al., 2012; Soden and Chen, 2010; Zhang et al., 2018). The loss of FMRP disrupts protein synthesis, which is linked to impaired synaptic plasticity, and hippocampal circuit dysfunction providing a mechanistic basis for the learning and memory deficits observed in FXS.

#### 3.3.2 Rett syndrome

Rett syndrome is a neurodevelopmental disorder caused by mutations in methyl-CpG binding protein 2 (MECP2), a methyl-CpG–binding transcriptional regulator (Tillotson and Bird, 2020). It predominantly affects females due to its X-linked inheritance and is characterized by developmental regression and progressive cognitive impairments. Individuals with Rett syndrome also exhibit impairments in learning, memory retention, and adaptive behaviors (Neul et al., 2010). Similar learning and memory deficits are also observed in animal models of Rett Syndrome. Mecp2308 (Mecp2 deficient) mice exhibit impaired hippocampus-dependent spatial memory, contextual fear memory, and social memory, which are associated with abnormalities in synaptic plasticity (Moretti et al., 2006; Tillotson and Bird, 2020). Rett syndrome is also associated with dysfunction of hippocampal and cortical networks involved in memory encoding and consolidation (Tillotson and Bird, 2020). Loss of MeCP2 disrupts excitatory and inhibitory balance, resulting in altered network synchronization and impaired information processing (Chao et al., 2010). Defects in glutamatergic and GABAergic circuit function have been reported in Rett models, suggesting that disrupted excitatory-inhibitory balance contributes to impaired memory-related network activity (Chao et al., 2007, 2010; Na et al., 2012).

The cognitive deficits observed in Rett syndrome are thought to result, at least in part, from disruption of MeCP2-dependent transcriptional regulation essential for neuronal maturation and synaptic function (Zhou et al., 2006; Na et al., 2012; Tillotson and Bird, 2020). Loss of MeCP2 alters the expression of genes required for these processes, resulting in widespread abnormalities in synaptic signaling and plasticity (Na et al., 2012; Sánchez-Lafuente et al., 2022; Tillotson and Bird, 2020). These molecular changes are accompanied by impaired hippocampal LTP and altered LTD in Mecp2-null mice, indicating disruption of the cellular mechanisms that support learning and memory (Asaka et al., 2006). Beyond these Hebbian forms of plasticity, MeCP2 deficiency also disrupts activity-dependent homeostatic synaptic scaling, further compromising the ability of neural circuits to maintain stable network activity (Qiu et al., 2012). Together, defects in activity-dependent transcription, synaptic strength, and excitatory–inhibitory balance provide a mechanistic framework linking MeCP2 dysfunction to memory impairment in Rett syndrome.

#### 3.3.3 Angelman syndrome

Angelman syndrome results from the loss of maternal *UBE3A* expression and is associated with severe impairments in learning, working memory, and long-term memory retention, often accompanied by deficits in associative learning (Williams et al., 2006; Bird, 2014). These cognitive impairments are associated with dysfunction of hippocampal circuits involved in memory encoding and consolidation and are accompanied by deficits in experience-dependent learning and memory formation in both patients and animal models (van Woerden et al., 2007; Weeber et al., 2003).

UBE3A encodes an E3 ubiquitin ligase that regulates synaptic protein turnover and activity-dependent synaptic function. Loss of maternal UBE3A also disrupts synaptic protein homeostasis and synaptic plasticity mechanisms, including long-term potentiation in hippocampal circuits, resulting in impaired experience-dependent memory formation (Sato and Stryker, 2010; Sun et al., 2015; Su et al., 2023). These disruptions contribute to abnormalities in information processing and hippocampal circuit function that impair memory formation (Greer et al., 2010; Sun et al., 2015; Weeber et al., 2003). These findings suggest that loss of UBE3A disrupts memory processing across multiple levels, linking impaired ubiquitin-mediated regulation of synaptic proteins and plasticity mechanisms to circuit dysfunction and deficits in learning and memory.

#### 3.3.4 Down syndrome

Down syndrome (DS) is the most prevalent genetic cause of ID and is characterized by a distinct memory profile (Godfrey and Lee, 2018; Lanfranchi et al., 2012). Individuals with DS show pronounced impairments in verbal working memory and episodic memory (Baddeley and Jarrold, 2007; Carney et al., 2013; Godfrey and Lee, 2018). These cognitive impairments are associated with structural and functional alterations in hippocampal–prefrontal circuits involved in memory encoding, consolidation, and retrieval (Koenig et al., 2021; Smigielska-Kuzia et al., 2011).

Underlying these cognitive impairments are alterations in synaptic development, neurotransmission, and synaptic plasticity that disrupt the formation and function of neural circuits required for memory processing (Cramer and Galdzicki, 2012; Koenig et al., 2021). Emerging evidence further suggests that DS-associated genes such as DSCR1 and DYRK1A regulate dendritic spine dynamics, synaptic development, and activity-dependent plasticity (Murphy et al., 2024; Wang et al., 2012). The interaction between DSCR1 and FMRP further implicates dysregulated local protein synthesis in DS pathophysiology (Wang et al., 2012).

Disruptions in Hebbian plasticity, including impaired hippocampal LTP, and alterations in excitatory–inhibitory balance have been implicated in the learning and memory deficits observed in DS models (Kleschevnicov et al., 2004; Kleschevnikov et al., 2012). The molecular and synaptic abnormalities in DS, including altered protein synthesis and synaptic plasticity, are thought to disrupt hippocampal–prefrontal circuit function and information processing, providing a mechanistic framework linking altered plasticity to memory dysfunction in Down syndrome.

#### 3.3.5 Tuberous sclerosis complex

Tuberous Sclerosis Complex (TSC) is caused by hyperactivation of mTOR signaling, leading to disrupted hippocampal synaptic plasticity and long-term potentiation, producing deficits in learning and memory (Ehninger et al., 2008). Human studies reveal hippocampal–prefrontal structural abnormalities that correlate with memory impairment, while recent work suggests persistent developmental synaptic dysregulation and circuit immaturity contribute to deficits in memory encoding and retrieval (Ridler et al., 2007; Feliciano, 2020; López-Aranda et al., 2024).

Individuals with TSC exhibit impairments in working memory, episodic memory, and memory precision, reflecting dysregulated mTOR-dependent plasticity (Ehninger et al., 2008; Tsai and Sahin, 2011). Recent evidence further suggests that cognitive dysfunction in TSC may exhibit sex-dependent characteristics, with female Tsc2+/– mice displaying object recognition memory deficits, whereas male mice show impairments in social memory, highlighting the importance of considering sex as a biological variable when investigating memory dysfunction in TSC (López-Aranda et al., 2024). Altered mGluR-dependent synaptic plasticity and excitatory–inhibitory imbalance further contribute to hippocampal hyperexcitability and circuit dysfunction, while homeostatic compensatory mechanisms appear insufficient to restore normal network activity (Bateup et al., 2011, 2013). The convergence of mTOR-dependent translational dysregulation, impaired synaptic plasticity, and circuit abnormalities is thought to underlie the memory deficits observed in TSC.

Across diverse genetic etiologies, memory impairment in ID converges on disruption of working memory capacity, executive control, and goal-directed retrieval, driven by altered synaptic plasticity, activity-dependent transcription, and neural circuit development (Alloway, 2010; Danielsson et al., 2010).

## 4 Conclusion

Memory dysfunction across neurodevelopmental disorders reflects multilevel disruptions spanning molecular signaling, synaptic plasticity, circuit coordination, and developmental experience. In ADHD, memory impairments arise primarily from altered regulation of prefrontal–striatal–cerebellar networks that support attention-dependent encoding and executive control, whereas in autism spectrum disorder (ASD), memory deficits often reflect impaired integration of episodic, sensory, and social information across distributed cortical–hippocampal circuits. In ID, disruptions in working memory capacity, executive control, and goal-directed retrieval arise from diverse genetic and developmental etiologies that affect activity-dependent transcription, translational regulation, protein homeostasis, synaptic plasticity, and circuit maturation. Together, this multiscale perspective positions memory as a unifying phenotype linking behavioral dysfunction with molecular signaling pathways, synaptic plasticity mechanisms, and circuit-level dysfunction across neurodevelopmental disorders. Evidence discussed throughout this review suggests that both Hebbian and homeostatic forms of plasticity contribute to memory-related circuit function, and disruption of these complementary processes may represent a common mechanism underlying cognitive dysfunction across neurodevelopmental disorders. A key unresolved question is whether memory dysfunction represents a primary driver across neurodevelopmental disorders or an emergent consequence of broader circuit instability. Addressing this will require longitudinal and circuit-resolved approaches linking molecular perturbations to behavioral outcomes across development.

An additional layer of complexity arises from sex-dependent differences in the behavioral, circuit, and molecular mechanisms underlying memory dysfunction across neurodevelopmental disorders. ASD exhibits a marked male bias in prevalence and may involve sex-specific alterations in gene expression, synaptic signaling pathways, and neural circuit development (Lai et al., 2015; Werling and Geschwind, 2013). Emerging evidence from TSC and DS models further suggests that males and females can exhibit distinct learning and memory phenotypes (Ahmed et al., 2021; López-Aranda et al., 2024). These differences may arise from interactions between genetic risk factors, sex chromosome complement, hormonal influences, and activity-dependent plasticity mechanisms that differentially shape synaptic function and circuit maturation (Lai et al., 2015; Werling and Geschwind, 2013). Understanding how biological sex modulates these processes will be important for explaining the heterogeneity of memory dysfunction and for developing more precise therapeutic strategies.

Moving forward, future research must move beyond single-gene or single-circuit approaches toward integrative models that link behavioral phenotypes with circuit-level and molecular mechanisms across development. Advances in neuroimaging, single-cell transcriptomics, and circuit-specific manipulation in animal models will allow more precise mapping of memory-relevant networks and their developmental trajectories. Emerging work on molecular signaling, non-coding RNAs, and experience-dependent plasticity highlights the dynamic nature of memory circuits and their potential for therapeutic modulation. Longitudinal and transdiagnostic approaches will be particularly valuable in identifying convergent pathways underlying memory dysfunction across ADHD, ASD, and ID. Such frameworks may enable early identification of at-risk individuals and guide targeted interventions that restore circuit balance and improve cognitive outcomes.
